# Health Boards in Italy

**Published:** 1887-03

**Authors:** 


Health Boards in Italy.—As it is proposed to re-organize the Health Department of Buffalo it will not be uninteresting to know that Italy has just enacted a law regulating the organization of municipal boards of health, as follows:

Section 1. Every municipality (*comune*) shall have a Board of Health whose duty it shall be to give advice concerning all matters pertaining to the public health, to suggest means of preventing disease, and to abate all nuisances which would tend to injure the public health.

Sec. 2. Every Municipal Board of Health shall be composed as follows:

One or two members of the Common Council—*consiglieri comunali.*

One, two or three physicians.

A pharmacist.
A veterinary surgeon.
A civil engineer. —*Il. Raccoglitore Med.*

---

THE Buffalo Board of Health has among its district physicians one who claims that the normal temperature of the human body is from 32° to 40° Reamur, that is 104° to 109° Fahrenheit. He claimed that he was not familiar with the Fahrenheit scale. We will state that he practices homœopathy and was not appointed under civil service rules.

